# Exploration of Race and Ethnicity, Sex, Sport-Related Concussion, Depression History, and Suicide Attempts in US Youth

**DOI:** 10.1001/jamanetworkopen.2022.19934

**Published:** 2022-07-01

**Authors:** Shawn R. Eagle, David Brent, Tracey Covassin, Robert J. Elbin, Jessica Wallace, Justus Ortega, Raymond Pan, Martina Anto-Ocrah, David O. Okonkwo, Michael W. Collins, Anthony P. Kontos

**Affiliations:** 1 University of Pittsburgh, Pittsburgh, Pennsylvania; 2 Michigan State University, East Lansing; 3 University of Arkansas, Fayetteville; 4 University of Alabama, Tuscaloosa; 5 Humboldt State University, Arcata, California

## Abstract

**Importance:**

Racial, ethnic, and sex disparities for concussion incidence and suicide attempts in youth have been reported, but data on the interaction of these variables in a large national data set are lacking. Understanding how race and ethnicity interact with sex and concussion to influence suicide attempts could yield critical information on the sociocultural impact of brain injury and mental health in US youth.

**Objective:**

To examine the associations of concussion history, race and ethnicity, and sex with reported suicide attempts among adolescents.

**Design, Setting, and Participants:**

This population-based cross-sectional cohort study used data from US Youth Risk Behavior Surveillance System (YRBSS) survey respondents between 2017 and 2019. Data were analyzed from May 2021 to January 2022.

**Exposures:**

Respondents reported sport- or recreation-related concussion (yes or no), depression (yes or no), and suicide attempt (yes or no) over the previous 12 months, along with race and ethnicity (categorized as American Indian or Alaska Native, Asian, Black, Hispanic/Latino, multiracial, Native Hawaiian or other Pacific Islander, and White), and sex (male or female).

**Main Outcomes and Measures:**

Two Chi-Square Automatic Interaction Detection (CHAID) decision tree models were built. The first was suicide attempt with depression history (SA-DEP), the second was suicide attempt without depression history (SA–NO DEP). CHAID uses risk factors (eg, number of concussions, race and ethnicity, sex) to divide the study sample into a series of subgroups that are nested within each other. Risk ratios (RRs) and 95% CIs were calculated for each subgroup to provide effect estimates.

**Results:**

A total of 28 442 youths aged up to 18 years (mean [SD] age, 14.6 [3.0] years; 14 411 [50.7%] female) responded to the survey. The CHAID decision trees revealed a complex interaction between race, sex, and concussion history for attempting suicide, which differed by depression history (overall accuracy, 84.4%-97.9%). Overall, depression history was the variable most strongly associated with SA (adjusted odds ratio, 11.24; 95% CI, 10.27-12.29). Concussion was the variable most strongly associated with SA-DEP (RR, 1.31; 95% CI, 1.20-1.51; *P* < .001). Black, Hispanic/Latino, or multiracial race and ethnicity were associated with increased risk for SA-DEP compared with others (RR, 1.59; 95% CI, 1.38-1.84; *P* < .001). American Indian or Alaska Native, Black, and Hispanic/Latino race and ethnicity were associated with increased risk for SA–NO DEP (RR, 1.89; 95% CI, 1.54-2.32; *P* < .001) compared with the remaining population.

**Conclusions and Relevance:**

These findings suggest that clinicians should consider race, ethnicity, and sex when evaluating the role of sport- or recreation-related concussion on suicide risk among US youth.

## Key Points

**Question:**

Is the interaction of race and ethnicity, sex, depression, and concussion associated with risk of suicide attempts in US youth?

**Findings:**

In this cohort study among 28 442 youth, a Chi-Square Automatic Interaction Detection model identified the combination of concussion history and Black, Hispanic/Latino or multiracial race and ethnicity yielded the highest risk of suicide attempt among youth with depression history. American Indian or Alaska Native, Black, or Hispanic/Latino race and ethnicity had the highest risk for reporting suicide attempt among youth without depression history.

**Meaning:**

These findings suggest that clinical phenotypes of race and ethnicity, sex, depression, and concussion history were useful in understanding risk for suicide attempts in US youth.

## Teaser

This cohort study examines associations of concussion history, race and ethnicity, sex, and depression with reported suicide attempts among US youth.

## Introduction

Up to 3.2 million adolescents experience a concussion in the US annually.^1^ *Concussion* is defined as a traumatically induced disturbance of brain function that involves a complex pathophysiological process that can affect physical, emotional, cognitive, and sleep-related function.^1^ Approximately 30% to 39% of adolescents with concussion experience prolonged symptoms that can impair daily functioning and overall well-being.^2,3^ In a sample of adolescents with persistent postconcussion symptoms, Chrisman et al^2^ reported that 14% of adolescents had thoughts of self-harm and 8% had suicidal ideation. Results from a meta-analytic review by Fralick et al^3^ involving nearly 7 million individuals from ages 8 to 58 years indicated that concussion history was associated with a 2-fold increased suicide risk.^3^ These findings highlight the association of concussion and suicide risk, but the role of other potential factors, including race and ethnicity, sex, and depression, in the association of concussion to risk for suicide attempts is not well described.

Racial and ethnic disparities exist in concussion knowledge,^4^ care-seeking,^5^ reporting,^4,6^ and management,^7^ and racial and ethnic disparities in mental health awareness, incidence of depression, and treatment are well known.^8,9,10,11^ Sex also plays a moderating role in concussion, depression, and suicide attempts, as female adolescents attempt suicide at significantly higher rates than males while males experience concussion at higher rates than females.^12,13,14,15^ It is difficult to estimate the rate at which concussions and/or suicide attempts occur for each race and ethnicity because of sample-size limitations of previous studies, differences among racial and ethnic groups in reporting behaviors, and discrepancies in the diagnosis of concussion among races and ethnicities.^5,14,16^ Using data from the Youth Risk Behavior Surveillance System (YRBSS), a biannual, serial survey given to US adolescents to track risk behaviors over time, a study by Xiao et al^12^ reported that Black adolescents had the largest increase in suicide attempts from 1991 to 2019 (80%), followed by American Indian or Alaska Native adolescents (70%). Although their study also examined suicide attempt trends by sex within racial and ethnic groups, it did not evaluate concussion history as a covariate.^12^ A study by Wangnoo et al^17^ used YRBSS data from a single state to assess the role of concussion with suicide behaviors in 2013, but only included race as a covariate when estimating suicidal behaviors. There is an urgent need to assess how these key demographic constructs (ie, race and ethnicity and sex) and depression history interact with concussion history in association with suicide attempts in a large, nationally representative data set, given the associations among these variables when analyzed separately across different studies.

The primary purpose of this study was to examine the associations of concussion history, race and ethnicity, and sex with reported suicide attempts among adolescents in the YRBSS between 2017 and 2019. Specifically, this study sought to (1) describe the rates of concussion history and suicide attempts within the previous year by race and ethnicity, stratified by sex; (2) use logistic regression models to assess the associations of concussion history, race and ethnicity, and sex with the likelihood of suicide attempt after adjusting for depression history; and (3) use a data-mining algorithm to evaluate the interaction of concussion history, race and ethnicity, and sex in the likelihood of suicide attempt after adjusting for depression history. We hypothesized that concussion history would be a significant moderator of the associations of race and ethnicity and sex with suicide attempts.

## Methods

This cross-sectional study was deemed exempt from review because data were deidentified and publicly available. All survey respondents had parental assent to participate. Findings are reported in accordance with the Strengthening the Reporting of Observational Studies in Epidemiology (STROBE) reporting guideline for cohort studies.

This study was a cross-sectional cohort analysis of children and adolescents who completed to the national Youth Risk Behavior Surveillance System (YRBSS) survey in 2017 and 2019. The YRBSS is a serial, biannual survey completed nationally during school hours and sponsored by the Centers for Disease Control and Prevention with the goal of quantifying health risk behaviors among a representative sample of US youth over time. Completion of the survey is voluntary and requires parental consent. In 2017, the YRBSS began including a concussion history (during the previous 12 months) item in its questionnaire. Full description of YRBSS methods are described extensively in the literature.^18^ The overall response rate was 60% in 2017 and 2019, consistent with previous years.^19^

The primary outcome was binary responses to the survey item “During the past 12 months, how many times did you actually attempt suicide?” Responses were recorded ordinally and recoded categorically (yes or no). Other variables of interest for the present study were “During the past 12 months, how many times did you have a concussion from playing a sport or being physically active?” and “During the past 12 months, did you ever feel so sad or hopeless almost every day for two weeks or more in a row that you stopped doing some usual activities?” Participants additionally self-reported race and ethnicity (ie, American Indian or Alaska Native, Asian, Black, Hispanic/Latino, multiracial, Native Hawaiian or other Pacific Islander, White); sex (ie, female or male); and age (ie, ≤12, 13, 14, 15, 16, 17, or 18 years).

### Statistical Analysis

Descriptive statistics were calculated for the entire unweighted sample. For this study, the outcomes analyzed were concussion in the past 12 months (yes or no) and suicide attempt in the last 12 months (yes or no). Female sex, concussion history, and race and ethnicity were included as exposures for all analyses. Race and ethnicity were separated (ie, American Indian or Alaska Native, Asian, Black, Hispanic/Latino, multiracial, and Native Hawaiian or Pacific Islander) with White serving as the reference group in all analyses. Missing data were observed for each variable (age, 0.54%; sex, 0.98%; race and ethnicity, 2.73%; depression history, 1.74%; suicide attempt, 25.4%; concussion history, 9.69%). Missing data were imputed once using the classification and regression tree algorithm via SPSS Modeler. Classification and regression tree was used owing to its advantages with nonparametric and categorical data, which are likely to be collinear.^20,21^ Univariate logistic regressions (LR) were conducted to evaluate the association between a single exposure (eg, age, depression history within the previous year, sex, ≥1 concussions in the previous year, race and ethnicity) and likelihood of at least 1 suicide attempt within the last year. A single multivariable forward stepwise LR was conducted to evaluate the adjusted association (adjusted odds ratios [aOR] and 95% CIs) of each exposure on reporting at least 1 suicide attempt within the last year (cutoff for inclusion: *P* < .05). To assess the interaction between concussion history, race and ethnicity, or sex and the reporting of at least 1 suicide attempts, a Chi-Square Automatic Interaction Detection (CHAID) model was built. CHAID is a data mining algorithm that uses nonlinear decision boundaries to generate a decision tree based on the identification of patterns among variables.^21^ The CHAID algorithm automatically assesses nominal variables (ie, race and ethnicity in the current study) and merges categories that are not statistically different using the χ^2^ test.^21^ The strongest factor associated with the outcome (ie, suicide attempt) becomes the first cutpoint in the tree, and each subgroup is then identified recursively further down the decision tree until no further splits can be made that are statistically significant.^20^ Furthermore, the use of the CHAID algorithm allows automatic detection of interactions between the exposures (such as with concussion history and race and ethnicity) and within the exposure (such as the 7 races and ethnicities) to assess their association with suicide attempts. Because depression is strongly associated with suicide attempts, the data set was stratified by depression history (yes or no) for CHAID analyses, resulting in 2 decision trees (suicide attempt with depression history [SA-DEP] and suicide attempt without depression history [SA–NO DEP]). Another advantages of the CHAID algorithm is the use of Bonferroni corrections at each level for multiple comparisons. The node with the highest risk of the outcome (ie, suicide attempt) at each level was compared with the overall population using relative risk ratios (RRRs) and 95% CIs. To cross-validate model accuracy, boosting was used to obtain a sequence of models to ensure the accuracy of predictions. A priori significance level for all analyses was 2-sided *P* < .05. Analyses were conducted using SPSS statistical software version 28.0.1 and SPSS Modeler version 18.1 (IBM). Data were analyzed from May 2021 to January 2022.

## Results

### Descriptive Statistics

A total of 28 442 youths (mean [SD] age, 14.6 [3.0] years; 14 411 [50.7%] females) responded to the YRBSS, including 3874 respondents (13.6%) with history of at least 1 concussion over the last year and 1904 respondents (6.7%) who reported a suicide attempt in the past year (Table 1). Of those who endorsed a suicide attempt, 1497 respondents (78.6%) also endorsed depression history in the previous year. Males reported higher rates of concussion (2125 males [15.3%]) than females (1749 females [12.0%]) but females reported higher rates of suicide attempt (1240 females [8.5%]) than males (664 males [4.8%]). Native Hawaiian or Pacific Islander youth reported the highest rate of concussion (42 respondents [22.7%]), while Asian youth reported the lowest rate (141 respondents [11.1%]). American Indian or Alaska Native youth reported the highest rate of suicide attempts (32 respondents [11.3%]), while Asian youth reported the lowest rate (62 respondents [4.9%]).

**Table 1.** Descriptive Statistics for the Overall Sample

Characteristic	Participants, No. (%)		
	Concussion (n = 3874)	Suicide attempt (n = 1904)	Total (N = 28 442)
Age, y			
≤12	515 (12.9)	282 (7.4)	3789 (13.3)
13	966 (13.7)	476 (6.7)	7059 (24.8)
14	1041 (13.9)	490 (6.6)	7469 (26.3)
15	879 (13.1)	424 (6.3)	6713 (23.6)
≥16	473 (13.9)	232 (6.8)	3412 (12.0)
Sex			
Male	2125 (15.3)	664 (4.8)	13 885 (48.8)
Female	1749 (12.0)	1240 (8.5)	14 411 (50.7)
Race			
American Indian or Alaska Native	56 (19.9)	32 (11.3)	282 (1.0)
Asian	141 (11.1)	62 (4.9)	1266 (4.5)
Black	685 (14.2)	347 (7.2)	4836 (17.0)
Hispanic/Latino	899 (13.4)	480 (7.2)	6685 (23.5)
Multiracial	199 (13.4)	143 (9.6)	1484 (5.2)
Native Hawaiian or other Pacific Islander	42 (22.7)	15 (18.1)	185 (0.7)
White	1852 (13.5)	825 (6.0)	13 704 (48.2)

Rates of concussion and suicide attempt were identified for each race and ethnicity group stratified by sex (Table 2). Among males of all races and ethnicities, American Indian or Alaska Native youth had the highest rate of concussion (32 respondents [23.2%]) and suicide attempts (19 respondents [11.7%]). Among females of all races and ethnicities, Native Hawaiian or Pacific Islander youth had the highest rate of concussion (20 respondents [25.6%]), while multiracial youth had the highest rate of suicide attempts (110 respondents [13.9%]). Males had higher rates of concussion than females for all races and ethnicities, except for Native Hawaiian or Pacific Islander (Table 2). Females had higher rates of suicide attempts than males for all races and ethnicities, except for American Indian or Alaska Native youth (Table 2).

**Table 2.** Descriptive Statistics of Concussion and Suicide Attempts by Sex and Race and Ethnicity Subgroups

Characteristic	Participants, No. (%)		
	Concussion	Suicide attempt	Total
**Male**			
American Indian or Alaska Native	32 (23.3)	19 (11.7)	163
Asian	70 (11.3)	27 (4.4)	618
Black	394 (16.7)	120 (8.9)	2358
Hispanic/Latino	492 (15.2)	161 (5.0)	3243
Multiracial	110 (16.0)	32 (4.7)	686
Native Hawaiian or other Pacific Islander	20 (20.2)	5 (5.1)	99
White	912 (14.6)	266 (4.3)	6243
**Female**			
American Indian or Alaska Native	17 (14.5)	13 (11.1)	117
Asian	71 (11.0)	35 (5.4)	644
Black	287 (11.6)	223 (9.0)	2466
Hispanic/Latino	404 (11.8)	312 (9.1)	3420
Multiracial	86 (10.9)	110 (13.9)	790
Native Hawaiian or other Pacific Islander	20 (25.6)	9 (11.5)	78
White	804 (12.1)	477 (7.2)	6643

### Logistic Regression Analyses

#### Univariate LR

The exposure most strongly associated with suicide attempt, in terms of variance accounted for, was depression history (*R*^2^ = 0.13; odds ratio [OR], 8.43; 95% CI, 7.53-9.44), followed by female sex (*R*^2^ = 0.01; OR, 1.81; 95% CI, 1.64-1.99), and concussion history (*R*^2^ = 0.004; OR, 1.59; 95% CI, 1.41-1.79). Race and ethnicity were also associated with reporting a suicide attempt (*R*^2^ = 0.004) (Table 3). American Indian or Alaska Native race and ethnicity was the racial and ethnic group with the highest risk of suicide attempt (OR, 2.00; 95% CI, 1.37-2.91), followed by multiracial (OR, 1.66; 95% CI, 1.38-2.01), Black (OR, 1.21; 95% CI, 1.06-1.37), and Hispanic/Latino (OR, 1.21; 95% CI, 1.07-1.36) compared with youth who identified as White (Table 3). Age (*R*^2^ = 0.003; *P* < .001) was also associated with suicide attempts in the previous year (OR, 0.96; 95% CI, 0.94-0.99).

**Table 3.** Univariable Logistic Regression Models to Identify Variables Associated With Reporting Suicide Attempt in the Previous Year

Characteristic	OR (95% CI)
Age	0.96 (0.94-0.99)
Depression history	8.43 (7.53-9.44)
Concussion history	1.59 (1.41-1.79)
Female sex	1.81 (1.64-1.99)
Race and ethnicity^a^	
American Indian or Alaska Native	2.00 (1.37-2.91)
Asian	0.80 (0.62-1.05)
Black	1.21 (1.06-1.37)
Hispanic/Latino	1.21 (1.07-1.36)
Multiracial	1.66 (1.38-2.01)
Native Hawaiian or other Pacific Islander	1.38 (0.81-2.35)

Abbreviation: OR, odds ratio.

^a^ Compared with White respondents.

#### Multivariable LR

The multivariable LR model including 28 422 youth was significant (*R*^2^ = 0.20; *P* < .001) (Table 4). The model included depression history in the previous year (adjusted OR [aOR], 11.24; 95% CI, 10.27-12.29), concussion history in the previous year (aOR, 1.33; 95% CI, 1.20-1.47), female sex (aOR, 1.23; 95% CI, 1.14-1.34), American Indian or Alaska Native race and ethnicity (aOR, 1.70; 95% CI, 1.18-2.45), multiracial identity (aOR, 1.46; 95% CI, 1.25-1.70), Hispanic/Latino race and ethnicity (aOR, 0.89; 95% CI, 0.81-0.98), and age (aOR, 0.96; 95% CI, 0.93-0.99) as variables associated with reporting a suicide attempt in the previous year.

**Table 4.** Multivariable Logistic Regression Model

Characteristic	aOR (95% CI)	*P* value
Age	0.96 (0.93-0.99)	.009
Depression history	11.24 (10.27-12.29)	<.001
Concussion history	1.33 (1.20-1.47)	<.001
Female sex	1.23 (1.14-1.34)	<.001
Race and ethnicity^a^		
American Indian or Alaska Native	1.70 (1.18-2.45)	.004
Hispanic/Latino	0.89 (0.81-0.98)	.01
Multiracial	1.46 (1.25-1.70)	.04

Abbreviation: aOR, adjusted odds ratio.

^a^ Compared with White respondents.

### CHAID Decision Trees

#### Decision Tree for SA-DEP

The CHAID decision tree for SA-DEP had an overall accuracy of 84.4% (Figure, A). Concussion history within the previous year was the variable most strongly associated with SA-DEP (RR, 1.31; 95% CI, 1.20-1.51; adjusted *P* < .001). Of respondents who reported at least 1 concussion in the previous year, reporting Black, Hispanic/Latino, or multiracial race and ethnicity was associated with increased risk for SA-DEP compared with the remaining population (RR, 1.59; 95% CI, 1.38-1.84; adjusted *P* < .001). Black, Hispanic/Latino or multiracial females had increased risk for SA-DEP compared with Black, Hispanic/Latino, or multiracial males (RR, 1.34; 95% CI, 1.00-1.77; adjusted *P* = .04). Of those who reported 0 concussions in the previous year, being female and American Indian or Alaska Native, multiracial, or Native Hawaiian or Pacific Islander was associated with increased risk for SA-DEP (RR, 1.52; 95% CI, 1.28-1.82; adjusted *P* < .001).

**Figure.**
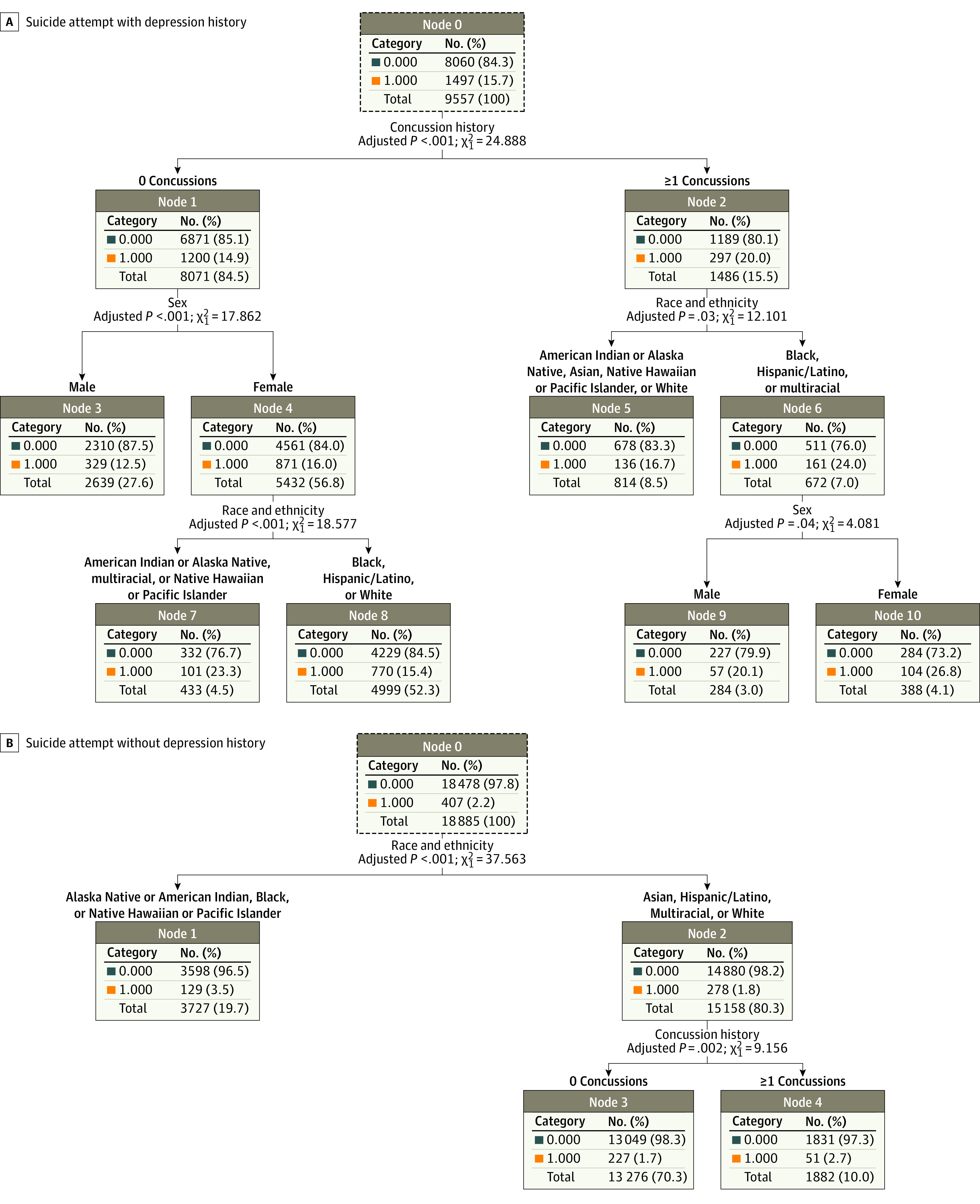
Chi-Square Automatic Interaction Detection Decision Trees to Assess the Interaction Among Concussion History, Sex, and Race and Ethnicity on Suicide Attempts A, Overall accuracy, 84.3%. B, Overall accuracy, 97.8%.

#### Decision Tree for SA–NO DEP

The CHAID Decision tree for SA–NO DEP had an overall accuracy of 97.9% (Figure, B). Race and ethnicity was the variable most strongly associated with SA–NO DEP, with American Indian or Alaska Native, Black, and Native Hawaiian or Pacific Islander races and ethnicities being associated with increased risk for SA–NO DEP (RR, 1.89; 95% CI, 1.54-2.32; adjusted *P* < .001) compared with the remaining population. Within the Asian, Hispanic/Latino, multiracial, and White cohort, reporting at least 1 concussions in the previous year was not associated with significantly increased risk for SA–NO DEP compared with those who reported no concussions (RR, 1.29; 95% CI, 0.97-1.73; adjusted *P* = .08).

## Discussion

In this cross-sectional study using data from the US YRBSS survey, a history of concussion was more strongly associated with suicide attempts among youth of specific underrepresented races and ethnicities, including American Indian or Alaska Native, multiracial identity, and Hispanic/Latino. Reporting depression in the previous year was the variable most strongly associated with reporting a suicide attempt in the previous year, with an aOR of 11.24. This highlights how critical depression screening is among US youth for preventing suicide attempts. After adjusting for depression history and age in a multivariable logistic regression model, race and ethnicity, concussion history, and female sex were associated with significantly increased odds of reporting suicide attempts in the previous year. The CHAID algorithm revealed 3 sociocultural and clinical phenotypes most strongly associated with suicide attempts in the previous year, based on endorsing depression or not. Among those who endorsed depression, 2 combinations had higher risk for reporting suicide attempts: (1) concussion history, female sex, and Black, Hispanic/Latino, or multiracial race/ethnicity and (2) no concussion history, female sex, and American Indian or Alaska Native, multiracial, or Native Hawaiian or Pacific Islander race and ethnicity. Among those not endorsing depressive symptoms in the previous year, individuals identifying as American Indian or Alaska Native, Black, or Hispanic/Latino had the highest risk for suicide attempt. Importantly, these analyses are only suggestive of an association between these variables. Causality cannot be determined owing to an inability to discern when depression, suicide attempt, and concussion history occurred in the previous year.

An advantage of the CHAID algorithm is its ability to automatically identify interactions by race and ethnicity, as well as the automatic detection of interactions by race and ethnicity, sex, and concussion history associated with suicidal behavior. Black, Hispanic/Latino, or multiracial youth with depression and past-year concussion history had 59% increased likelihood of suicide attempt. Being female within the Black, Hispanic/Latino or multiracial cohort was associated with 33% increased risk compared with males in that cohort. Female American Indian or Alaska Native, multiracial, or Native Hawaiian or Pacific Islander youth with depression history, but without concussion history, had 52% increased likelihood of a suicide attempt. Race and ethnicity was the primary variable associated with likelihood of suicide attempt in those without depression history in the previous year: identifying as American Indian or Alaska Native, Black, or Hispanic/Latino was associated with 89% increased risk. Unfortunately, this study could not parse out the temporal relationships among concussion, depression, and suicide attempt. We therefore cannot determine causality among these variables. Psychiatric sequelae after concussions are common in adolescents, but there are limited prospective data on concussion and the association with suicidal behavior among youth. Some retrospective studies suggest that risk of suicide is increased 2- to 3-fold beginning soon after the concussion.^22^ Critically, a concussion in combination with a preinjury psychiatric disorder or a postinjury, novel psychiatric disorder increased odds of suicide 17.9-fold and 11.9-fold, respectively.^22^

Sociodemographic factors can be major contributors to stress across a variety of domains, which contributes to risk for depression and suicide.^14^ Specifically, racialized stress related to living in the US, combined with historical inequality and inequities in health care, could be substantial contributors to a feeling of overall stress.^23^ The multivariable logistic regression analyses suggested that American Indian or Alaska Native adolescents had higher odds of suicide attempt (70%) than White adolescents. These increased odds were higher than any other race or ethnicity in this study and remained after adjusting for depression, concussion history, and female sex. Furthermore, among male respondents, American Indian or Alaska Native youth had 3.4% higher rates of concussion than the next-highest race or ethnicity and 5.7% higher rates of suicide attempt than the next-highest race or ethnicity (both Native Hawaiian or Pacific Islander youth). It is well known that American Indian or Alaska Native youth experience disproportionately greater mental health concerns and suicide behavior.^24,25^ For example, a study by Whitbeck et al^26^ reported an increase in lifetime rates for at least 1 disorder from the *Diagnostic and Statistical Manual of Mental Disorders* (Fourth Edition), from 25.6% to 44.8% in American Indian or Alaska Native youth from 8 different reservations. However, only 1 prior study, to our knowledge, has investigated the associations of concussion history among American Indian or Alaska Native individuals with mental health.^27^ The study by Nelson et al^27^ examined adults and found that American Indian or Alaska Native individuals had a higher lifetime prevalence of traumatic brain injury (TBI) than expected (8%-26%), and those individuals with lifetime TBI had 2-fold increased likelihood of a mood or anxiety disorder compared with those without TBI history.^27^ Future research should investigate the temporal associations among these variables in different racial and ethnic groups to understand how one variable may interact with the other.

Black and Hispanic/Latino adolescents had equally higher odds of reporting a suicide attempt compared with White adolescents over the previous year. Black and Hispanic/Latino youths are more likely to grow up in economically disadvantaged communities within the US compared with White adolescents and are more likely to experience multidimensional poverty than White adolescents.^23^ Black adolescents represent the largest increase in prevalence of suicide attempts between 1991 and 2019, according to a study using YRBSS data.^12^ A study by Vega et al^28^ reported that Black and Hispanic/Latino boys had lower self-esteem, higher depressive symptoms, and were more often belittled by teachers and parents than White boys and that these were risk factors for suicidal behavior. Furthermore, other studies have reported that compared with White adolescents, Hispanic/Latino adolescents had higher odds of experiencing major depressive episodes but were less likely to receive adequate mental health care than White adolescents.^29,30^ Both Black and Latino individuals have higher underdiagnosed rates of depression compared with White individuals, which is perhaps related to sociocultural norms of emotional expression and access to or availability of appropriate mental health services.^12^

There is limited research investigating differences in concussion incidence and reporting between athletes of different races and ethnicities. A study by Wallace et al^5^ reported no difference between White and Black high school athletes in reported concussions. In a large epidemiological study using the National Electronic Injury Surveillance System (NEISS) data set over a 10-year period, including more than 11 million pediatric emergency department visits, Black athletes were 30% less likely to be diagnosed with a sport-related concussion compared with White athletes.^16^ Moreover, a study by Wallace and Mannix^31^ also reported similar results in US adolescents: Black youth were 34% less likely to have an emergency department visit for a concussion compared with White youth. This gap in care may represent a meaningful opportunity for clinicians to link Black and Hispanic/Latino individuals with a potential brain injury to the appropriate health care resources. This study found relatively similar rates of reported concussion history among Asian, Black, Hispanic/Latino, multiracial, and White individuals (11.1%-14.2%), while American Indian or Alaska Native (19.9%) and Native Hawaiian or Pacific Islander (22.7%) reported concussions in the previous year at disproportionately higher rates. While prior work has shed light on this topic, it has largely been limited by comparing one race or ethnicity (ie, Black/African Americans) with White individuals, limiting our understanding of how concussion among other minoritized groups.

This study involved analysis of a large, nationally representative data set including more than 50% females. Seven different races and ethnicities were assessed, adding representation to the literature and nuance to other approaches, which typically assess Black, Hispanic/Latino, and White races and ethnicities and merge the remaining races and ethnicities into an other category. Identifying individuals who attempt suicide both with and without depression history may yield critical information to researchers and clinicians about the unique impact of social determinants of health within the context of concussion and suicide behaviors. For example, race and ethnicity was the primary factor that differentiated youth who attempted suicide without depression history, with American Indian or Alaska Native, Black, and Native Hawaiian or Pacific Islander youth having 89% increased risk for suicide attempt compared with other races and ethnicities. Attempting suicide in the absence of reported depression history may suggest how impactful race and ethnicity are on mental well-being and prospective outlook. However, it is critical for clinicians to understand that reporting a 2-week period of depressive symptoms in the past year was the overwhelmingly strongest risk factor associated with a suicide attempt compared with other variables in this study. Screening for depressive symptoms routinely is critical to ensure the safety of adolescents who are members of racial and ethnic minority groups.

### Limitations

This study has some limitations. The cross-sectional nature of the YRBSS survey data precludes our ability to draw causal or temporal inferences with regards to the associations of race and ethnicity, sex, and concussion exposures with the outcome of suicide attempt. Variables known to be associated with suicide attempts (eg, psychiatric diagnosis, stress, adverse life experiences) were not included in the YRBSS data set. Other variables that were not available in the national YRBSS data set, such as socioeconomic status, parents’ level of education, insurance status, and other medical history, could have been relevant to our primary outcomes. The absence of these variables may help explain why the combination of age, sex, depression history, concussion history and race and ethnicity only accounted for 20% of the variation in reporting a suicide attempt in the previous year. Clearly, future work must consider other variables to improve our understanding of the variation related to suicide attempts in US youth. Given that the YRBSS survey was voluntary and required parental permission, the data set may have been subject to response bias. Survey responses were also self-reported by adolescents and could not be verified with medical records. Missing data were identified and imputed once with the Classification and Regression Tree algorithm using SPSS Modeler. Imputing data once (compared with multiple times) may lead to imprecision; however, there is disagreement on this issue in the literature.^32^ The software used for this study did not allow for multiple imputation. These models should be externally validated in future YRBSS data sets.

## Conclusions

In this cohort study of US YRBSS data, we identified an interaction among concussion, race and ethnicity, and female sex that increased risk for reporting at least 1 suicide attempt among youth with depression in the previous year by 59%. Furthermore, American Indian or Alaska Native, Black, and Hispanic/Latino race and ethnicity were associated with 89% increased risk for reporting at least 1 suicide attempt in the previous year for youth without depression. Clinicians should consider how concussion and depression may uniquely influence suicide attempts based on race and ethnicity and biological sex. A prospective study of premorbid mental health, concussion, postmorbid mental health, and suicidal behaviors in a racially and ethnically diverse cohort of US adolescents is warranted.
